# When do tripdoublet states fluoresce? A theoretical study of copper(II) porphyrin

**DOI:** 10.3389/fchem.2023.1259016

**Published:** 2023-11-10

**Authors:** Xingwen Wang, Chenyu Wu, Zikuan Wang, Wenjian Liu

**Affiliations:** ^1^ Qingdao Institute for Theoretical and Computational Sciences, Shandong University, Qingdao, China; ^2^ Max-Planck-Institut für Kohlenforschung, Mülheim an der Ruhr, Germany

**Keywords:** tripdoublet, fluorescence, photophysics, spin-adapted TDDFT, SDSPT2

## Abstract

Open-shell molecules rarely fluoresce, due to their typically faster non-radiative relaxation rates compared to closed-shell ones. Even rarer is the fluorescence from states that have two more unpaired electrons than the open-shell ground state, since they involve excitations from closed-shell orbitals to vacant-shell orbitals, which are typically higher in energy compared to excitations from or out of open-shell orbitals. States that are dominated by the former type of excitations are known as tripdoublet states when they can be described as a triplet excitation antiferromagnetically coupled to a doublet state, and their description by unrestricted single-reference methods (e.g., U-TDDFT) is notoriously inaccurate due to large spin contamination. In this work, we applied our spin-adapted TDDFT method, X-TDDFT, and the efficient and accurate static-dynamic-static second order perturbation theory (SDSPT2), to the study of the excited states as well as their relaxation pathways of copper(II) porphyrin; previous experimental works suggested that the photoluminescence of some substituted copper(II) porphyrins originate from a tripdoublet state, formed by a triplet ligand *π* → π* excitation antiferromagnetically coupled with the unpaired d electron. Our results demonstrated favorable agreement between the X-TDDFT, SDSPT2 and experimental excitation energies, and revealed noticeable improvements of X-TDDFT compared to U-TDDFT, not only for vertical excitation energies but also for adiabatic energy differences. These suggest that X-TDDFT is a reliable tool for the study of tripdoublet state fluorescence. Intriguingly, we showed that the aforementioned tripdoublet state is only slightly above the lowest doublet excited state and lies only slightly higher than the lowest quartet state, which suggests that the tripdoublet of copper(II) porphyrin is long-lived enough to fluoresce due to a lack of efficient non-radiative relaxation pathways; an explanation for this unusual state ordering is given. Indeed, thermal vibration correlation function (TVCF)-based calculations of internal conversion, intersystem crossing, and radiative transition rates confirm that copper(II) porphyrin emits thermally activated delayed fluorescence (TADF) and a small amount of phosphorescence at low temperature (83 K), in accordance with experiment. The present contribution is concluded by a few possible approaches of designing new molecules that fluoresce from tripdoublet states.

## 1 Introduction

**FIGURE 1:**
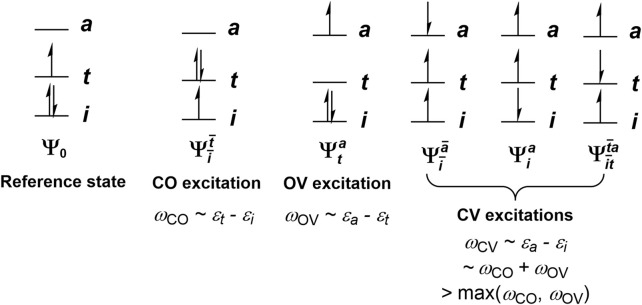
Schematic depictions of closed-open (CO), open-vacant (OV), and closed-vacant (CV) excitations, and their approximate excitation energies as predicted from restricted open-shell Kohn-Sham (ROKS) orbital energy differences.

Fluorescence, while ubiquitous in organic and organometallic molecules, is in most cases observed in closed-shell systems. It is well-known that introducing an open-shell impurity, such as dioxygen (Schmidt, 2006), a stable organic radical (Green et al., 1990) or a transition metal ion (Varnes et al., 1972), frequently quenches the fluorescence of a closed-shell molecule (Evans, 1957). One reason of this phenomenon is that the addition of an unpaired electron to a system typically introduces additional low-lying states, in particular charge transfer states that involve an electron exciting from or out of the new open-shell orbital (O). Moreover, while spin-conserving single excitations of a singlet reference determinant from closed-shell (C) to vacant-shell (V) orbitals, hereafter termed CV excitations following our previous works (Li and Liu, 2010; Li et al., 2011; Li and Liu, 2011; Wang et al., 2020), give rise to *n*_C_ *n*_V_ singlet excited states and *n*_C_ *n*_V_ triplet excited states (where *n*_C_ and *n*_V_ are the number of closed-shell an vacant-shell orbitals, respectively), with an *M*_*S*_ = 1/2 doublet determinant one obtains 2*n*_C_ *n*_V_ excitations that are mixtures of doublets and quartets (the $\Psi_{\bar{i}}^{\bar{a}}$ and $\Psi_{i}^{a}$ determinants in Figure 1; here orbitals without overbars denote *α* orbitals, and those with overbars denote *β* ones). They can be linearly combined to make *n*_C_ *n*_V_ pure doublet states, but the other linear combination remains a mixture of doublet and quartet:

$$\Psi_{singdoublet}=\frac{1}{\sqrt{2}}\left(\Psi_{i}^{a}+\Psi_{\bar{i}}^{\bar{a}}\right),$$ (1)

$$\Psi_{mixed}=\frac{1}{\sqrt{2}}\left(\Psi_{i}^{a}-\Psi_{\bar{i}}^{\bar{a}}\right).$$ (2)

In spin-adapted TDDFT methods, the latter are spin-adapted to give 2*n*_C_ *n*_V_ pure doublet states and *n*_C_ *n*_V_ quartet states, by mixing with the *n*_C_ *n*_V_ spin flip-up excitations from the *M*_*S*_ = −1/2 component of the reference determinant, i.e., the $\Psi_{\bar{i}t}^{\bar{t}a}$ determinants in Figure 1 (Li and Liu, 2010; Li et al., 2011; Li and Liu, 2011):

$$\Psi_{tripdoublet}=\frac{1}{\sqrt{6}}\left(-\Psi_{i}^{a}+\Psi_{\bar{i}}^{\bar{a}}+2\Psi_{\bar{i}t}^{\bar{t}a}\right),$$ (3)

$$\Psi_{quartet}=\frac{1}{\sqrt{3}}\left(\Psi_{i}^{a}-\Psi_{\bar{i}}^{\bar{a}}+\Psi_{\bar{i}t}^{\bar{t}a}\right).$$ (4)

Note that both the “singdoublets” and “tripdoublets” are pure doublet states. While the singdoublets Eq. 1 (which we called the CV(0) states in our previous works (Li and Liu, 2010; Li et al., 2011; Li and Liu, 2011)) are direct analogs of singlet excited states out of a singlet reference, the tripdoublets Eq. 3 (CV(1) states) do not have analogs in closed-shell systems, and create extra spin-allowed non-radiative relaxation pathways compared to when the reference determinant is singlet. This further contributes to the short excited state lifetimes of doublet systems. As a consequence, doublet molecules (and open-shell molecules in general) are rarely fluorescent.

Still, there exist open-shell molecules that do fluoresce, which have found applications in, e.g., organic light-emitting diodes (OLEDs) (He et al., 2019; Gao et al., 2023). However, their fluorescence usually originates from an excited state that has only one unpaired electron, i.e., a CO or OV excited state (where CO stands for a single excitation from a closed-shell orbital to an open-shell one; similar for OV), instead of a CV excited state. This can be partly rationalized by approximating the excitation energies of the system by orbital energy differences. Under this approximation, there is at least one CO state and one OV state below any given CV state, since the lowest CV excitation energy is the sum of the excitation energies of a CO state and an OV state (Figure 1). Therefore, the lowest CV state tends to be a rather high excited state of the system, and thus usually has more energetically accessible non-radiative relaxation pathways than the low-lying CO and OV states do, rendering fluorescence from CV states especially hard to achieve. To counter this, one may try to inhibit the non-radiative relaxation of the CV state to lower excited states. However, the sheer number of non-radiative relaxation pathways that one would have to inhibit poses a great challenge for designing an open-shell molecule that fluoresces from a CV state. Alternatively, one may design a system where the orbital energy difference approximation fails dramatically, allowing the lowest CV state to become the first excited state, or only slightly higher than the first excited state. In this case, the fluorescence from the CV state only needs to compete with the intersystem crossings (ISCs) to the lowest quartet state(s) and the internal conversion (IC) to the ground state and lower doublet excited state(s), which are the only energy downhill non-radiative relaxation pathways available to the CV state. In particular, note that when the CV excitations shown in Figure 1 linearly combine to give singdoublets, tripdoublets and quartets via Eqs 3, 4, there is an energy splitting that usually places the quartet below the tripdoublet, and the tripdoublet below the singdoublet; while the former is a consequence of Hund’s rule, the latter can be rationalized by applying Hund’s rule after neglecting the coupling of the open-shell orbital to the closed-shell and vacant-shell ones. This gives tripdoublets a much greater chance than singdoublets for emitting fluorescence with an appreciable quantum yield. Nevertheless, the singdoublet-tripdoublet splitting appears to be small in general, compared to the orbital energy difference that one would have to overcome, which can amount to several eVs. Hence, even the fluorescence from tripdoublets proves to be scarce.

**FIGURE 2:**
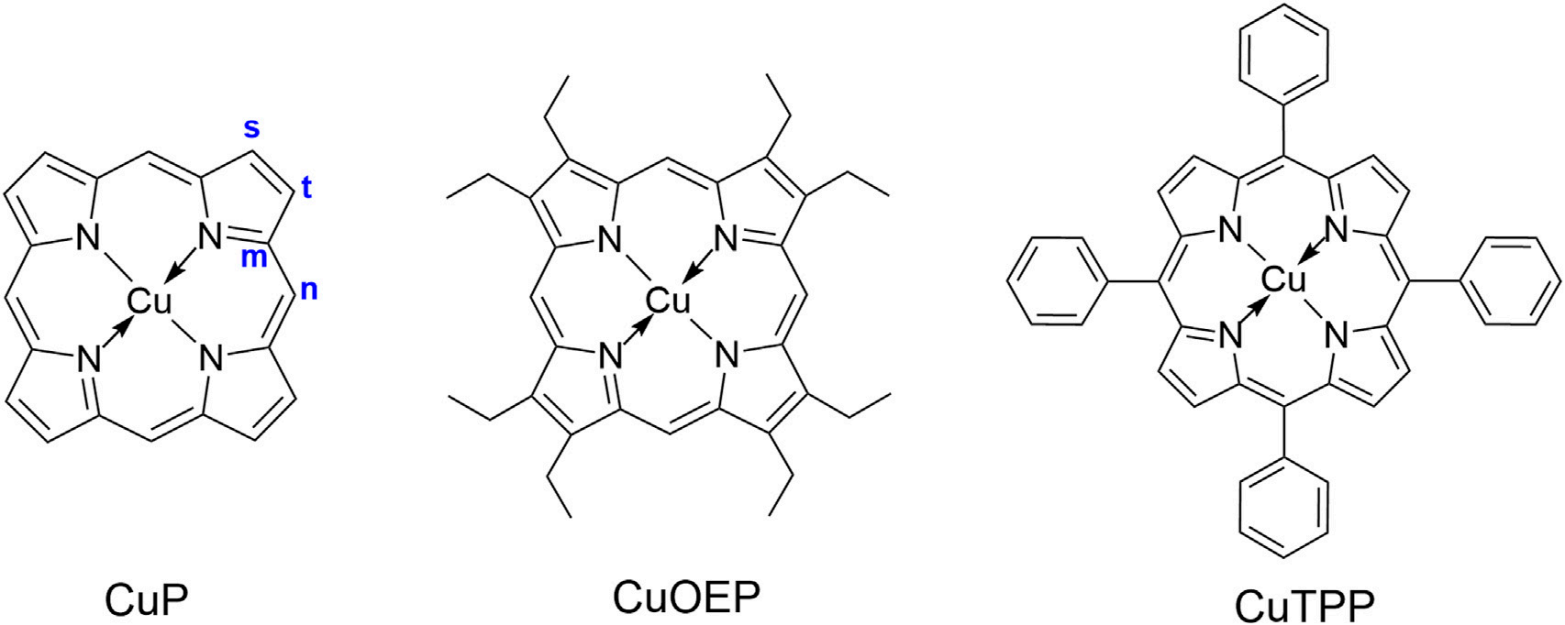
Molecular structures of CuP, CuOEP and CuTPP.

The present paper represents a preliminary attempt to unveil some of the factors that enable an open-shell molecule to fluoresce from a tripdoublet state, via a case study of copper(II) porphyrin complexes. Copper(II) porphyrin complexes (Figure 2), like most porphyrin complexes, show two intense visible absorption bands near 390–420 nm and 520–580 nm (Gouterman, 1959; Eastwood and Gouterman, 1969); they are conventionally termed the B and Q bands, respectively. Eastwood and Gouterman (1969) studied the luminescence of copper(II) porphyrin molecules in the solid state by exciting their Q bands, suggesting that the emission may originate from one of the two low-lying *π* → π* states, ^2^T or ^4^T (here the 2, 4 represent the overall spin multiplicity of the complex, and T denotes that the “local” spin multiplicity of the porphyrin ring is triplet). They speculated that a rapid equilibrium may exist between the ^2^T and ^4^T states. The equilibrium ratio of these two states is largely dependent on the energy gap (Δ*E*_DQ_) between them and the temperature, via the Boltzmann distribution. The radiative transition from the ^2^T state to the ground state is spin-allowed, making it much faster than the phosphorescence from the ^4^T state. Thus, when Δ*E*_DQ_ is small and the temperature is high, the experimentally observed rapid emission is predominantly from the ^2^T state. Conversely, when Δ*E*_DQ_ is large and the temperature is low, a slow emission attributed to the phosphorescence of the ^4^T state was observed instead, due to the concentration of the ^4^T state largely overwhelming that of the ^2^T state. Thus, molecules such as copper 2,3,7,8,12,13,17,18-octaalkylporphyrin (CuOAP), which possess small Δ*E*_DQ_ values, exhibit luminescence primarily in the form of fluorescence from the ^2^T state at liquid nitrogen temperature, whereas copper 5,10,15,20-tetraphenylporphyrin (CuTPP) with a larger Δ*E*_DQ_ mainly undergoes phosphorescence from the ^4^T state at the same temperatures. The unsubstituted copper porphyrin (CuP) is the most interesting of all, as pure phosphorescence was observed at low temperatures (35 K), which gradually gives way to fluorescence when the temperature was elevated, eventually giving pure fluorescence at 143 K (Bohandy and Kim, 1980). Similar results have been obtained by following works with different techniques and/or solvents (Magde et al., 1974; Kobayashi et al., 2008).

The simple and intuitive picture has since been supplemented by subsequent works, which also excited the B band, and proposed that charge transfer (CT) states may play an important role in the relaxation of the initial bright state to the essentially dark ^2^T state. Yan and Holten (1988) investigated the excited state relaxation processes of CuTPP and CuOEP at different temperatures and in different solvents, proposing possible pathways involving intermediate states that are probably ligand-to-metal CT (LMCT) states. This is supported by the gas-phase mass spectrometry experiments by Ha-Thi et al. (2013), although the precise composition of the CT state remains uncertain. Understanding the excited-state relaxation pathways of copper porphyrins is crucial for gaining insights into their photophysical processes and controlling their optical properties. In particular, whether any other excited state(s) lie below the ^2^T state may have a profound influence on whether the ^2^T state fluoresces or not, as follows from Kasha’s rule. Meanwhile, the energy gap of the ^2^T and ^4^T states is important for the relative concentration of the two states, and therefore the relative intensities of fluorescence from the ^2^T state and the phosphorescence from the ^4^T state, i.e., whether the experimentally observed luminescence should be attributed to fluorescence or phosphorescence, or both.

Despite the importance of tripdoublet fluorescence and the long history of experimental studies of copper porphyrins, accurate computational studies of this system prove to be difficult, as traditional unrestricted single-reference methods like U-TDDFT suffer from severe spin contamination issues, leading to systematically underestimated excitation energies. In particular, tripdoublet states are the worst scenario for U-TDDFT, as when the reference state itself is not spin-contaminated, the errors of the U-TDDFT ⟨*S* ^2^⟩ values reach the theoretical maximum of singly excited states, i.e., 2, for tripdoublet states (Li and Liu, 2010; Li et al., 2011; Li and Liu, 2011; Li and Liu, 2016a; Wang et al., 2020). While multireference methods trivially solve the spin contamination problems, it is notoriously difficult to obtain an accurate multireference description of the electronic structure of metalloporphyrins, due to the complex interplay between static and dynamic correlation. In this study, we employed the methods developed by our group, namely, X-TDDFT (Li and Liu, 2011; Wang et al., 2020) and SDSPT2 (Liu and Hoffmann, 2014; Lei et al., 2017) (static-dynamic-static second-order perturbation theory), to address these challenges and provide a rational description of the photophysical processes in CuP. As the first rigorous spin-adapted TDDFT method (Li and Liu, 2011), X-TDDFT gives spin-adapted excited states even when the reference state is open-shell, thereby generally giving better excitation energies, as well as better transition matrix elements involving the excited states. The recent development of the analytic gradient of X-TDDFT (Wang et al., 2020) allowed us to use X-TDDFT for excited state geometry optimization and seminumerical Hessian calculations as well. For vertical excitation calculations, we could afford to use SDSPT2, which also served as a reference for benchmarking X-TDDFT and U-TDDFT.

## 2 Computational details

All DFT, TDDFT, and SDSPT2 calculations were performed using a development version of the Beijing Density Functional (BDF) package (Liu et al., 1997; Liu et al., 2003; Liu et al., 2004a; Liu et al., 2004b; Zhang et al., 2020). Geometry optimizations were conducted using the PBE0 (Adamo and Barone, 1999; Ernzerhof and Scuseria, 1999) functional and x2c-SVPall (Pollak and Weigend, 2017) basis set in the gas phase, including Grimme’s D3 dispersion correction (Grimme et al., 2010; Grimme et al., 2011), as implemented in the BDF software; relativistic effects were considered at the spin-free exact two component (sf-X2C) level (Liu et al., 2003; Li et al., 2014b; Liu et al., 1995; Liu et al., 2004b; Liu et al., 2004a). For transition metal complexes (especially when excited states are considered), the choice of the optimum functional may not be obvious. Herein, TDDFT calculations were performed using four different functionals [BP86 (Perdew, 1986a; Perdew, 1986b; Becke, 1988), B3LYP (Becke, 1993; Stephens et al., 1994), PBE0 and *ω*B97X (Chai and Head-Gordon, 2008)] in conjunction with the x2c-TZVPall (Pollak and Weigend, 2017) basis set, followed by benchmarking the results against SDSPT2 and experimental results, and the PBE0 functional was chosen based on its satisfactory and uniform accuracy (see Section 3.1 for details). The TDDFT/x2c-TZVPall energies differ from the TDDFT/x2c-SVPall ones by within 0.04 eV for ligand excited states (^2^T_1_, ^2^T_2_, ^2^S_1_, ^2^S_2_), within 0.14 eV for d-d excited states, and up to 0.29 eV for CT states. Based on this, we used TD-PBE0/x2c-SVPall in all rate constant calculations that involve only the ligand excited states; for rate constants that involve the d-d states (where even TDDFT/x2c-TZVPall predicts wrong state orderings), we calculated the adiabatic excitation energies of the corresponding states using SDSPT2 vertical absorption energies plus the adiabatic/vertical excitation energy differences calculated at the TD-PBE0/x2c-SVPall level, and calculated all the other requisite quantities with TD-PBE0/x2c-SVPall. The orbital diagrams were drawn and visualized with VMD v.1.9.4 (Humphrey et al., 1996), using cube files generated with the help of Multiwfn v.3.8(dev) (Lu and Chen, 2012).

The calculations of ISC rate constants were conducted by the ESD module of the ORCA program, version 5.0.4 (Neese, 2012; Neese, 2018; Neese et al., 2020; Neese, 2022), using the thermal vibration correlation function (TVCF) method based on a multimode harmonic oscillator model. Other rate constants involved in the excited state relaxation process were calculated by the MOMAP package, version 2022A (Peng et al., 2007; Niu et al., 2008; Niu et al., 2020), again using the TVCF method and a harmonic approximation of the potential energy surfaces. The default parameters of the two programs were used in all TVCF calculations, except for the “tmax” parameter in the MOMAP calculations (which controls the propagation time of the TVCF), which was set to 3,000 fs. For the ^2^T_1_ →^2^dd_3_ IC rate constant, the tmax = 3,000 fs calculation suffered from numerical instabilities, so we chose to use tmax = 1,500 fs instead. All necessary transition matrix elements, including the transition dipole moments, non-adiabatic coupling matrix elements (NACMEs) (Li et al., 2014a; Li and Liu, 2014; Wang et al., 2021), spin-orbit coupling matrix elements (SOCMEs) (Li et al., 2013; Li et al., 2014a; Li et al., 2014b), as well as the seminumerical Hessians necessary for the TVCF calculations, were calculated by BDF. Note however that all NACMEs were computed by U-TDDFT instead of X-TDDFT, since the theory of X-TDDFT NACMEs has not been developed yet; similarly, geometry optimization and frequency calculations of the ^4^T_1_ state were performed at the unrestricted Kohn-Sham (UKS) level, which is justified by the small spin contamination (⟨*S* ^2^⟩ deviation < 0.1) of this state. The ALDA0 noncollinear exchange-correlation (XC) kernel (Li and Liu, 2012) was used in all spin flip-up Tamm-Dancoff approximation (TDA) calculations (i.e., calculation of quartet states from a doublet reference), which has proven essential for obtaining correct spin state splittings (Li and Liu, 2016b). Duschinsky rotation was considered whenever applicable. The Herzberg-Teller effect was only considered while calculating the radiative relaxation rates, but not the ISC rates, due to program limitations; however this should not change the qualitative conclusions of this paper, since all ISC processes whose Franck-Condon contributions are negligible or zero are expected to contribute negligibly to the photophysics of CuP. Although we have implemented the interface for calculating the Herzberg-Teller effect of phosphorescence by BDF and MOMAP, the computation of the geometric derivatives of the doublet-quartet transition dipole moments by finite differences proved to be numerically ill-behaved, as the *M*_*S*_ = ±1/2 and *M*_*S*_ = ±3/2 microstates of the ^4^T state mix strongly when the geometry is perturbed; note that this phenomenon seems to be related to the involvement of quartet states, since we have never observed similar behavior in triplet phosphorescence rate calculations. We thus estimated the total phosphorescence rate by assuming that the ratios of the Franck-Condon and Herzberg-Teller rates are the same for fluorescence and phosphorescence. This treatment is justified by the observation that the geometries and vibrational frequencies of the ^2^T_1_ and ^4^T_1_ states are very similar.For the SDSPT2 calculations, we employed the x2c-TZVPall basis set for the Cu and N atoms, and x2c-SVPall for the remaining atoms. The active space of the SDSPT2 calculations was selected through the iCAS (imposed automatic selection and localization of complete active spaces) method (Lei et al., 2021) using x2c-SVPall for all atoms, followed by projecting onto the aforementioned mixed basis, and the orbitals were optimized using the iCISCF [iterative configuration interaction (iCI)-based multiconfigurational self-consistent field (SCF) theory] method (Guo et al., 2021), which provided a reference wavefunction for the SDSPT2 calculation. An active space of CAS (13, 14) was used in this study. The B-band, Q-band and CT states involved in the excited state relaxation process mainly involve the Cu 3d and 4d orbitals, plus the four porphyrin *π* orbitals of the Gouterman four-orbital model (Gouterman, 1959), making a minimal active space of CAS (13, 14). The chosen active space thus properly describes the primary excited states of interest for investigation. Expanding the active space further would result in unnecessary computational overhead without providing additional insights. All SDSPT2 calculations reported herein include the Pople correction.

## 3 Results and discussion

### 3.1 Absorption process

As is well-known, density functionals generally have difficulties with simultaneously describing local excitation (LE) and CT states with good accuracy. Since we could only afford to do the geometry optimizations and frequency calculations under the DFT and TDDFT levels, a suitable functional that qualitatively reproduces the SDSPT2 excitation energies has to be chosen by comparing the TDDFT vertical absorption energies of a few common functionals with SDSPT2 data. B3LYP and PBE0 are generally common choices for the excited states of metalloporphyrins, and BP86 is often used to optimize their ground-state structures. Pure functionals usually tend to underestimate excitation energies, but empirically, their description of the Q band (an LE state) is better than many hybrid functionals, as will be confirmed by our calculation results. As CT states are involved in the relaxation process of the excited states of copper porphyrin, range-separated hybrid functionals (which provide good descriptions of CT states in general) may prove to be suitable as well. These considerations gave a list of four representative functionals, BP86, B3LYP, PBE0 and *ω*B97X, that were subjected to benchmark calculations.

**FIGURE 3:**
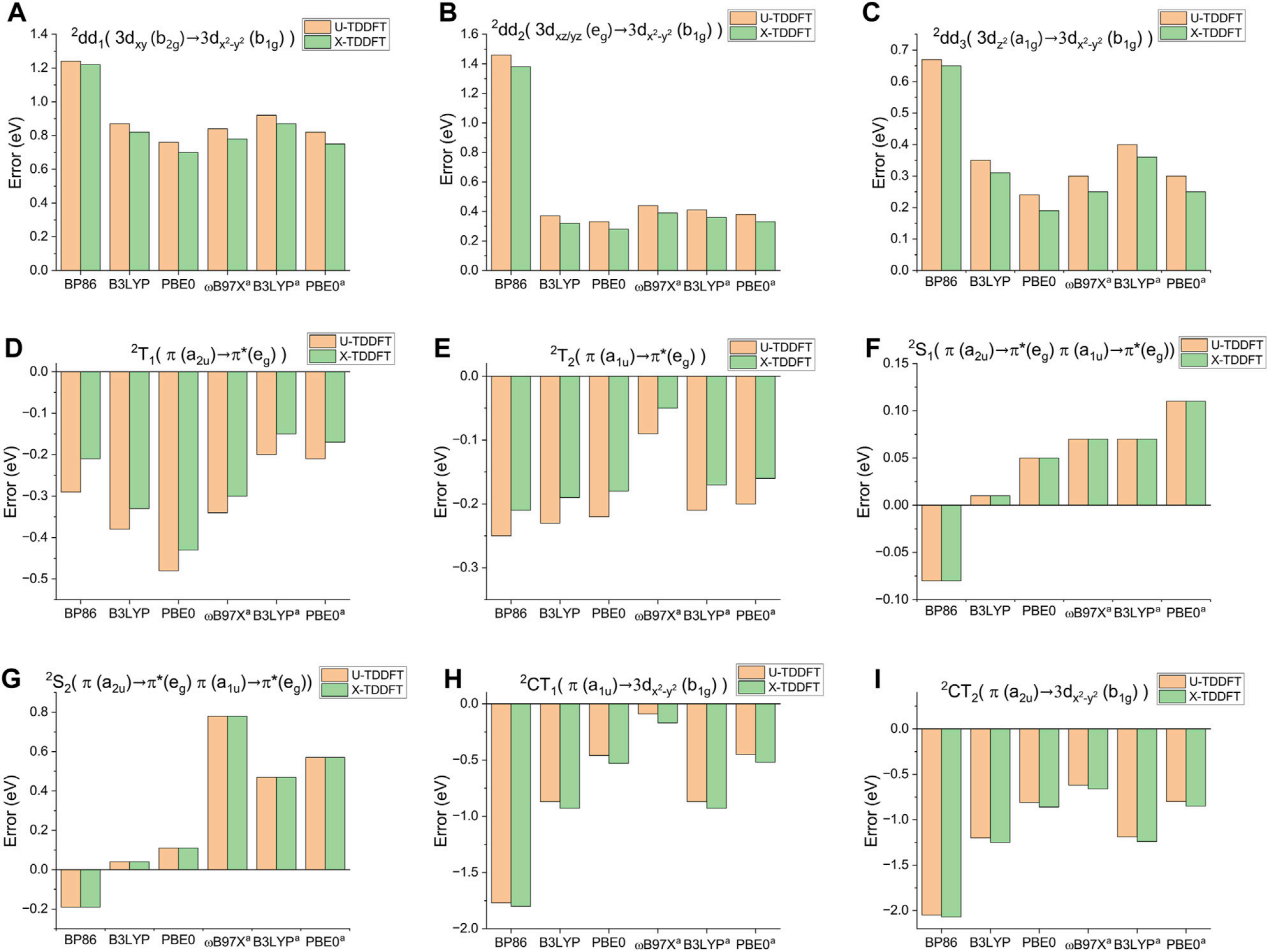
Errors of different excited states of CuP, computed by different functionals with the x2c-TZVPall basis set, with respect to SDSPT2 values. ^*a*^TDA.

Different functionals display distinct behaviors for the excitation energies of CuP compared to the results obtained from SDSPT2, as shown in Figure 3. The two characteristic absorption bands of the porphyrin molecule correspond to the ^2^S_1_ (Q band) and ^2^S_2_ (B band) states, which are the only bright states of most porphyrin complexes in the visible region. They are also the only excited states for which accurate experimental vertical absorption energies are available: in benzene they have been measured as 2.25 and 3.15 eV, respectively (Eastwood and Gouterman, 1969). Moreover, the absorption energy of the ^2^T_1_ state has been measured by fluorescence excitation spectra experiments, but only for certain substituted porphyrins: for example, the ^2^T_1_ absorption energy of CuEtio (Etio = etioporphyrin I) was measured in *n*-octane as 1.81 eV, while the emission energy from the same state in the same solvent was 1.79 eV (Eastwood and Gouterman, 1969). Assuming that the Stokes shift of the ^2^T_1_ state is independent of the porphyrin substituents, and combined with the experimental emission energy of the ^2^T_1_ state of CuP in the same solvent (1.88 eV) (Eastwood and Gouterman, 1969), we obtain an estimate of the experimental ^2^T_1_ absorption energy of CuP as 1.90 eV. Gratifyingly, the SDSPT2 excitation energies of all three states agree with the experimental values to within 0.3 eV, which is typical of the accuracy of SDSPT2 (Song et al., 2022) and confirms the suitability of SDSPT2 as a benchmark reference for CuP. The B3LYP functional performs better for the two bright states ^2^S_1_ and ^2^S_2_ than the other functionals (Figure 3), with results closer to the SDSPT2 calculations, suggesting its suitability for localized excitations in the porphyrin system. However, it (as well as the pure functional BP86) performs poorly in describing the dark charge transfer (CT) states, significantly underestimating their energies, as expected. In contrast, the range-separated functional *ω*B97X shows better agreement with the CT states compared to SDSPT2 results, but its description of the ^2^S_2_ state is rather poor, with energies notably higher than the SDSPT2 results. The PBE0 functional represents a compromise between the two classes of functionals and provides more accurate overall descriptions of the LE and CT states, giving results closer to the SDSPT2 calculations. In particular, PBE0 gives the best predictions of all the d-d excitation energies, although a significant overestimation is still seen for the lowest d-d state, ^2^dd_1_. Considering the overall performance in describing different states, we chose to use PBE0 for the remaining part of the present study.

The ^2^S_1_ and ^2^S_2_ states are almost spin-adapted states with minimal spin contamination, even at the U-TDDFT level (Table 1), since they are dominated by singdoublet excitations. As shown in Figure 3, both X-TDDFT and U-TDDFT provide similar descriptions for these two states; note however that functionals with large amounts of HF exchange generally overestimate the excitation energies of these two states, especially ^2^S_2_. At the TDDFT levels, the CT states are dominated by CO-type excitations (from *π* to 3 $d_{x^{2}-y^{2}}$ ), which are also spin-adapted. Both U-TDDFT and X-TDDFT show comparable performance in describing the CT states. However, both methods display large errors compared to SDSPT2 for the CT states. Table 1 presents the excitation energies and the corresponding dominant excited state compositions, computed at the ground state structure of CuP. It can be observed that the CT states are predominantly composed of double excitations, which cannot be described by the present TDDFT calculations. Despite this, functionals with large amounts of HF exchange still perform notably better, as is generally expected for CT states. The ^2^T_1_ and ^2^T_2_ states correspond to tripdoublet excitations (from *π* to π*), and they suffer from significant spin contamination at the U-TDDFT level, since instead of pure doublets, U-TDDFT can only describe these tripdoublet states as a heavy mixture of doublets and quartets, e.g.:

ΨT12U-TDDFT≈−13ΨT12X-TDDFT+23ΨT14,MS=1/2X-TDDFT, (5)

**TABLE 1:** The SDSPT2/x2c-TZVPall(Cu,N)/x2c-SVPall(C,H) excitation energies (in eV) computed at the sf-X2C-PBE0/x2c-SVPall ground state structure of CuP, along with the corresponding excited state compositions.

State	ΔE	Δ⟨S^2^⟩	Dominant transitions
^2^dd_1_	1.85	0.0019	3d_*xy*_ → 3 $d_{x^{2}-y^{2}}$ 88.0%
^2^dd_2_	2.02	0.0022	3d_*xz*_/3d_*yz*_ → 3 $d_{x^{2}-y^{2}}$ 87.8%
^2^dd_3_	2.13	0.0016	3 $d_{z^{2}}\;\to$ 3 $d_{x^{2}-y^{2}}$ 88.0%
^2^T_1_	2.19	1.9994	*π*(a_2*u*_) → π*(e_*g*_) 87.1%
^2^T_2_	2.32	1.9968	*π*(a_1*u*_) → π*(e_*g*_) 86.7%
^2^S_1_	2.43	0.0031	*π*(a_1*u*_) → π*(e_*g*_) 56.9%, *π*(a_2*u*_) → π*(e_*g*_) 36.1%
^2^S_2_	3.41	0.0115	*π*(a_2*u*_) → π*(e_*g*_) 52.5%, *π*(a_1*u*_) → π*(e_*g*_) 31.8%
^2^CT_1_	3.31	0.0066	[*π*(a_1*u*_) → Cu 3 $d_{x^{2}-y^{2}}$ (b_1*g*_) + Cu 3d_*xz*_/3d_*yz*_(e_*g*_) → π*(e_*g*_)] 65.6%
			*π*(a_1*u*_) → Cu 3 $d_{x^{2}-y^{2}}$ (b_1*g*_) 25.8%
^2^CT_2_	3.51	0.0086	[*π*(a_2*u*_)/*π*(a_2*u*_) → Cu 3 $d_{x^{2}-y^{2}}$ (b_1*g*_) + Cu 3d_*xz*_/3d_*yz*_(e_*g*_) → π*(e_*g*_)] 61.7%
			*π*(a_2*u*_) → Cu 3 $d_{x^{2}-y^{2}}$ (b_1*g*_) 21.6%

Δ⟨S^2^⟩: difference of the excited state’s ⟨S^2^⟩ value with the ground state ⟨S^2^⟩, computed at the U-TD-PBE0/x2c-TZVPall level. Transitions in square brackets represent double excitations.

As follows from Eqs 2–4. U-TDDFT thus systematically underestimates the excitation energies of the ^2^T_1_ and ^2^T_2_ states, since the energies of quartets are in general lower than the corresponding tripdoublets, as discussed in the Introduction. In Section 3.3 we will also see that part of the underestimation is due to the failure of U-TDDFT to reproduce the energy degeneracy of Ψ(T14,MS=1/2) and Ψ(T14,MS=3/2) . On the other hand, X-TDDFT avoids spin contamination through implicitly incorporating extra double excitations necessary for spin-adapting the tripdoublet states (Eq. 3), and therefore performs systematically better than U-TDDFT for all the functionals studied herein. The improvements of the excitation energies (∼0.05 eV) may seem small, but have profound influences on the magnitude and even the sign of the ^2^T_1_–^4^T_1_ gap, and therefore on the ratio of fluorescence and phosphorescence emission, as will be detailed in Section 3.3. Finally, X-TDDFT also improves upon the U-TDDFT excitation energies of the d-d excited states, despite that the latter are almost free from spin contamination.Already from the calculated absorption energies, one can draw some conclusions about the photophysical processes of CuP. The vertical absorption energy differences between the ^2^T_1_ and ^2^S_1_ states, as well as between the ^2^S_2_, ^2^CT_1_ and ^2^CT_2_ states, are very small (0.1–0.2 eV). Therefore, once CuP is excited to the bright ^2^S_1_ state by visible light, the molecule is expected to undergo a cascade of ultrafast IC processes, all the way till the doublet state, ^2^T_1_. While the fate of the ^2^S_2_ state is not immediately obvious from the excitation energies, it is well-known that the S_2_ states of porphyrin complexes typically undergo ultrafast IC to the S_1_ state on the hundred fs to 1 ps timescale (Bräm et al., 2019), suggesting that the ^2^S_2_ state of CuP will also quickly relax to the ^2^T_1_ state via ^2^S_1_, even without the presence of ^2^CT_1_; the presence of ^2^CT_1_ creates a further intermediate energy level between ^2^S_1_ and ^2^S_2_ and is expected to accelerate the IC further. The availability of an ultrafast IC cascade also means the ISC from these high-lying excited states are probably unimportant, especially considering that copper is a relatively light element. These findings are in qualitative agreement with the experimental observation that the ^2^S_2_ states of substituted copper(II) porphyrins relax to the ^2^T_1_ states in gas phase through a two-step process via the intermediacy of a CT state, with time constants 65 fs and 350–2,000 fs, respectively, depending on the substituents (Ha-Thi et al., 2013); the fact that the CT →^2^T_1_ IC is slower than the ^2^S_2_ → CT IC is consistent with the trend of our calculated energy gaps. In solution, the ^2^S_1_ state of Cu(II) protoporphyrin IX dimethyl ester was known to relax to ^2^T_1_ within 8 ps (Kobayashi et al., 2008), and for CuTPP as well as CuOEP the same relaxation was also found to occur within the picosecond timescale (Magde et al., 1974). Recently, the decay rates of the ^2^S_1_ state were measured as 50 fs and 80 fs for CuTPP and CuOEP, respectively, in cyclohexane (Bräm et al., 2019). The ^2^S_1_ state lifetime of CuP itself was also estimated, although indirectly from the natural width of the 0–0 peak of the Q band, as 30 fs (Noort et al., 1976). Quantitative computation of these IC rates is however beyond the scope of the paper, as the narrow energy gaps and possible involvement of conical intersections probably necessitate nonadiabatic molecular dynamics simulations. Nevertheless, a ^2^S_2_ →^2^CT_1_ →^2^S_1_/^2^T_2_ →^2^T_1_ IC pathway can still be tentatively proposed based on the energy ordering alone. Besides, there are also three d-d excited states with energies slightly lower (within 0.34 eV) than the ^2^T_1_ state. There is thus the possibility that the ^2^T_1_ state may further relax to either one of these three d-d states, which we will return to later. Finally, it is worth noting that the use of the accurate SDSPT2 method, as opposed to TDDFT, is crucial for obtaining a reliable estimate of the qualitative trend of the excited state energies. BP86 predicts that the CT states lie below the ^2^T states, leading to a qualitatively wrong IC pathway; *ω*B97X, on the other hand, grossly overestimates the energy of ^2^S_2_ and would underestimate its tendency to undergo IC to the CT states (Figure 3). While B3LYP and PBE0 predict reasonable excited state orderings, they still underestimate the energies of the CT states due to the presence of double excitation contributions, which cannot be correctly described under the adiabatic TDDFT framework.

### 3.2 Analysis of the equilibrium geometry of the ^2^T_1_ state

Since all higher lying excited states are predicted to convert to ^2^T_1_ over a short timescale, to study the luminescence of CuP [and probably also other Cu(II) porphyrin complexes bearing alkyl or aryl substituents, given that these substituents do not change excitation energies drastically (Eastwood and Gouterman, 1969)], it should suffice to study the radiative and non-radiative processes starting from the ^2^T_1_ state. Unlike the three ^2^dd states, whose Franck-Condon emissions are symmetry forbidden, the emission from the ^2^T_1_ state is symmetry allowed, and existing experimental results unequivocally point to a ligand state luminescence, rather than luminescence from a d-d excited state. Therefore, accurately predicting the equilibrium geometry of the ^2^T_1_ state is crucial for subsequent studies.

Some selected bond lengths for the optimized ground state and excited state structures are provided in Table 2. The difference in ground state bond lengths between the UKS and ROKS methods is extremely small ( < 0.0001 Å), as can be seen from their root mean square deviation (RMSD), which can be attributed to the extremely small UKS spin contamination of the ground state of CuP $\left({\left\langle S^{2}\right\rangle }_{PBE0}=0.7532\right)$ . The doubly degenerate ^2^T_1_ state, which belongs to the doubly degenerate E_*u*_ irreducible representation (irrep) under the D_4*h*_ group, undergoes Jahn-Teller distortion to give a D_2*h*_ structure, where two of the opposing Cu-N bonds are elongated but the corresponding pyrrole rings remain almost intact, while the other two Cu-N bonds are almost unchanged but the corresponding pyrrole rings exhibit noticeable deformation. The U-TDDFT and X-TDDFT bond lengths of the ^2^T_1_ state show larger deviations than the UKS and ROKS ground state ones, with the largest deviation exceeding 0.001 Å [the C-C (mn) bond], which is also reflected in the RMSD values. However, the structure differences are still small on an absolute scale. This suggests that the coupling of the unpaired Cu(II) d electron and the porphyrin triplet is weak, so that a reasonable tripdoublet state geometry is obtained even if this coupling is described qualitatively incorrectly (as in U-TDDFT). By contrast, our previous benchmark studies on small molecules (where the coupling between unpaired electrons is much larger) revealed that X-TDDFT improves the U-TDDFT bond lengths by 0.01–0.05 Å on average, depending on the functional and the molecule (Li and Liu, 2011; Wang et al., 2020).

**TABLE 2:** The equilibrium bond lengths (in $\overset{{}^{\circ}}{A}$ ) of the ground state (^2^S_0_) and the first doublet excited state (^2^T_1_) of the CuP molecule.

State	Cu-N	C-N	C-C (mn)^a^	C-C (mt)^a^	C-C (st)^a^
UKS					
^2^S_0_	2.0148	1.3652	1.3881	1.4410	1.3611
U-TDDFT					
^2^T_1_	2.0190	1.3683	1.4155	1.4145	1.3885
	2.0416	1.3674	1.3842	1.4447	1.3584
ROKS					
^2^S_0_	2.0148	1.3652	1.3881	1.4410	1.3612
X-TDDFT					
^2^T_1_	2.0198	1.3681	1.4152	1.4149	1.3886
	2.0413	1.3668	1.3853	1.4442	1.3590
RMSD^b^	0.00002				
RMSD^c^	0.00102				

^a^ See Figure 2 for the labeling of atoms.

^b^ The RMSD $\left(\overset{{}^{\circ}}{A}\right)$ between the optimized ^2^S_0_ state structures obtained using UKS and ROKS.

^c^ The RMSD $\left(\overset{{}^{\circ}}{A}\right)$ between the optimized ^2^T_1_ state structures obtained using U-TDDFT and X-TDDFT.

### 3.3 Relaxation processes of the ^2^T_1_ state

**FIGURE 4:**
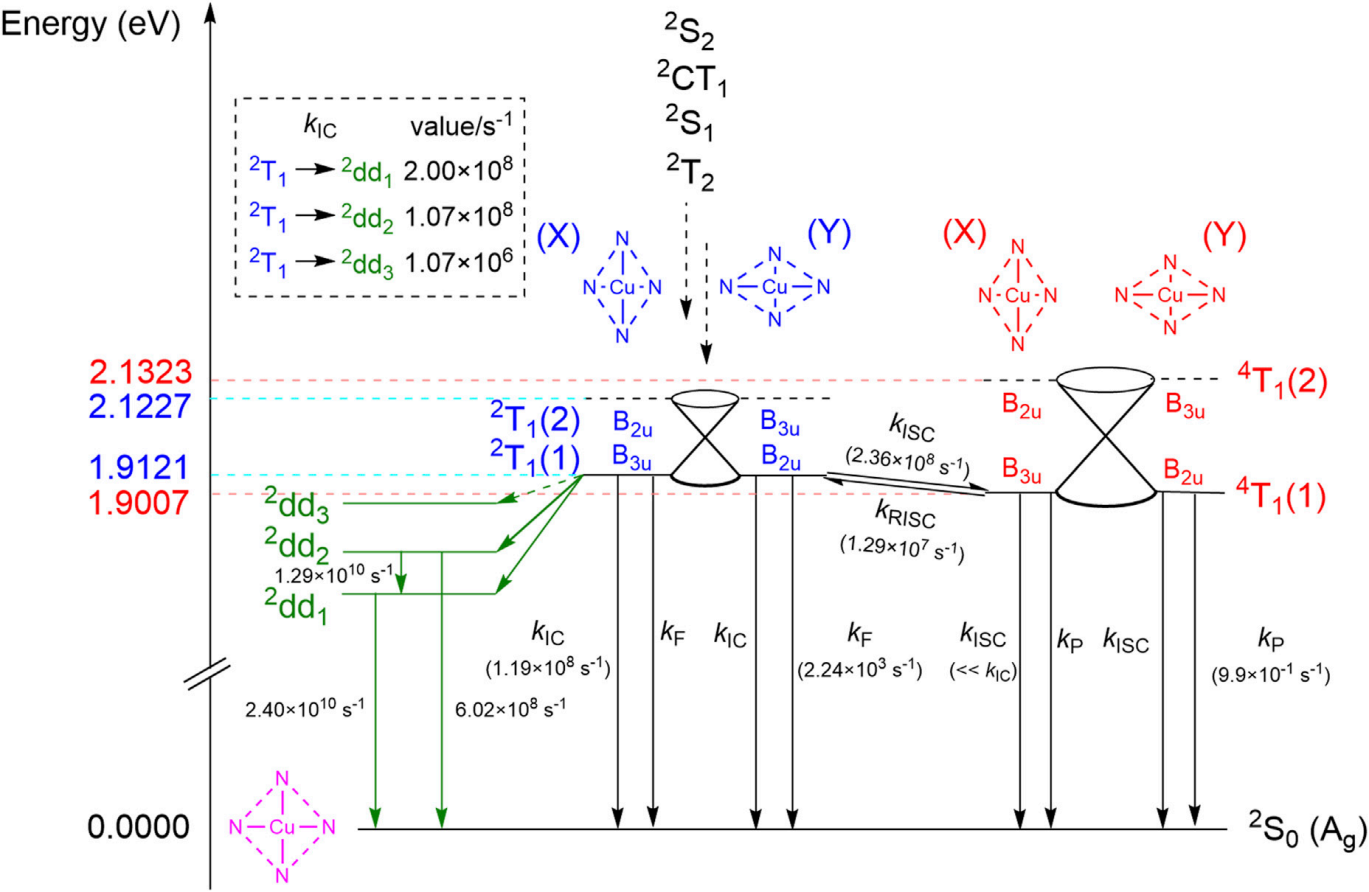
Radiative and non-radiative relaxation pathways of the ^2^T_1_ state. Both the ^2^T_1_ and ^4^T_1_ states are splitted by the Jahn-Teller effect to give two adiabatic states, labeled (1) and (2). Each of the (1) states have two equivalent D_2*h*_ equilibrium structures, labeled (X) and (Y). The (2) states do not have equilibrium structures and are connected with the corresponding (1) states via conical intersections. The X-TDA(doublet)/U-TDA(quartet) adiabatic excitation energies of the (1) states, as well as the energies of the (2) states at the equilibrium geometries of their corresponding (1) states, are shown on the left. The transition rates are calculated at 83 K in the gas phase. The forward and reverse ISC rates between ^2^T_1_(1)(X) and ^4^T_1_(1)(Y) are equal to those between ^2^T_1_(1)(Y) and ^4^T_1_(1)(X) by symmetry, but the former ISC processes are omitted for clarity. Transition rates that are obviously equal by symmetry reasons are shown only once. Transitions out of the ^2^dd_3_ state are not shown since the quantum yield of the ^2^dd_3_ state is expected to be negligible based on the small ^2^T_1_ →^2^dd_3_ IC rate.

As revealed by the above analyses, the relaxation process from high-lying excited states to the ^2^T_1_ state is rapid, and the only energetically accessible relaxation pathways are the radiative (fluorescence) and non-radiative (IC) relaxations from ^2^T_1_ to the ground state ^2^S_0_, the IC from ^2^T_1_ to one of the lower ^2^dd states, as well as the ISC from ^2^T_1_ to ^4^T_1_. The ^4^T_1_ state can furthermore convert back to the ^2^T_1_ state through reverse ISC (RISC), or relax to the ground state via radiative (phosphorescence) or non-radiative (ISC) pathways (Figure 4). While the RISC from ^4^T_1_ to the ^2^dd states is also possible, they are expected to be much slower than the spin-allowed ^2^T_1_ →^2^dd IC processes, and are therefore neglected in the present study.

Before we discuss the quantitative values of transition rates, we first analyze the relevant electronic states from the viewpoint of point group symmetry. The equilibrium structures of the ^2^T_1_ and ^4^T_1_ states are both distorted owing to the Jahn-Teller effect, and possess only D_2*h*_ symmetry, compared to the D_4*h*_ symmetry of the ground state equilibrium structure. The implications are two-fold: the double degeneracy of the ^*n*^T_1_(*n* = 2 or 4) state at the D_4*h*_ geometry (where they both belong to the E_*u*_ irrep) is lifted to give two adiabatic states, hereafter termed the ^*n*^T_1_(1) and ^*n*^T_1_(2) states, respectively, where ^*n*^T_1_(1) is the state with the lower energy; and the potential energy surface of the ^*n*^T_1_(1) state has two chemically equivalent D_2*h*_ minima, ^*n*^T_1_(1)(X) and ^*n*^T_1_(1)(Y), where different pairs of Cu-N bonds are lengthened and shortened (see the schematic depictions in Figure 4). Although ^*n*^T_1_(1)(X) and ^*n*^T_1_(1)(Y) are on the same adiabatic potential energy surface, their electronic wavefunctions represent different diabatic states, as they belong to the B_3*u*_ and B_2*u*_ irreps, respectively. The ^*n*^T_1_(1)(X) structure is diabatically connected to ^*n*^T_1_(2)(Y) (i.e., the ^*n*^T_1_(2) state at the equilibrium structure of ^*n*^T_1_(1)(Y)) via a D_4*h*_ conical intersection, while ^*n*^T_1_(1)(Y) is diabatically connected to ^*n*^T_1_(2)(X) via the same conical intersection. Thus, the ^*n*^T_1_(2)(X) and ^*n*^T_1_(2)(Y) states are expected to undergo ultrafast IC from the D_4*h*_ conical intersection, to give the ^*n*^T_1_(1)(Y) and ^*n*^T_1_(1)(X) states as the main products, respectively. The direct transition from ^*n*^T_1_(2) to states other than ^*n*^T_1_(1) can therefore be neglected. From the irreps of the electronic states, we conclude that certain ISC transitions are forbidden by spatial symmetry. These include the transitions between ^2^T_1_(1)(X) and ^4^T_1_(1)(X), between ^2^T_1_(1)(Y) and ^4^T_1_(1)(Y), and between any one of the ^4^T_1_(1) structures and ^2^S_0_. All IC and radiative transitions, plus the ISC transitions between ^2^T_1_(1)(X) and ^4^T_1_(1)(Y) as well as between ^2^T_1_(1)(Y) and ^4^T_1_(1)(X), are symmetry allowed. While symmetry forbidden ISC processes can still gain non-zero rates from the Herzberg-Teller effect, we deem that the rates are not large enough to have any noticeable consequences. On one hand, the two symmetry forbidden ISC pathways between the ^2^T_1_(1) and ^4^T_1_(1) states are overshadowed by the two symmetry allowed ones, so that the total ISC rate between ^2^T_1_(1) and ^4^T_1_(1) is undoubtedly determined by the latter alone. The ISC from ^4^T_1_(1) to ^2^S_0_, on the other hand, has to compete with the IC process from ^2^T_1_(1) to ^2^S_0_ in order to affect the quantum yield or the dominant relaxation pathway of the system noticeably, but the latter process is both spin-allowed and spatial symmetry-allowed, while the former is forbidden in both aspects. We therefore neglect all ISC rates whose Franck-Condon contributions are zero by spatial symmetry.

We then calculated the rate constants for all transitions between ^2^T_1_, ^4^T_1_ and ^2^S_0_ whose rates are non-negligible, by the TVCF method. The rates (Figure 4) were calculated at 83 K, the temperature used in the quantum yield studies of Eastwood and Gouterman (1969); the latter studies gave a luminescence quantum yield of 0.09, in a solvent mixture of diethyl ether, isopentane, dimethylformamide and ethanol. The accurate treatment of solvation effects is however complicated and beyond the scope of the paper, so that all transition rates were computed in the gas phase. Our calculated *k*_ISC_ from ^2^T_1_ to ^4^T_1_ (2.36 × 10^8^s^−1^) is slightly smaller than the total IC rate from ^2^T_1_ to ^2^S_0_ as well as to the three ^2^dd states (4.27 × 10^8^s^−1^), suggesting that treating the ^2^T_1_ and ^4^T_1_ states as a rapid equilibrium [as in, e.g., Ake and Gouterman (1969) and Bohandy and Kim (1980)] is not justified at least in the gas phase. The IC from ^2^T_1_ to ^2^dd_3_ is two orders of magnitudes slower than other non-radiative relaxation pathways of ^2^T_1_, and is therefore not considered viable in the remaining discussions. At 83 K, the RISC from ^4^T_1_ to ^2^T_1_ is 11% of the forward ISC rate. Both rates are in favorable agreement with the experimental values of Cu(II) protoporphyrin IX dimethyl ester in benzene, *k*_ISC_ = 1.6 × 10^9^s^−1^ and *k*_RISC_ = 5.6 × 10^8^s^−1^, at room temperature (Kobayashi et al., 2008). Our computed ISC and RISC rates give a ^2^T_1_-to-^4^T_1_ equilibrium concentration ratio of 1:9.0 when all IC processes are neglected, but our kinetic simulation shows that the steady state concentration ratio is 1:50.7 when the latter are considered, further illustrating that treating the ^2^T_1_-^4^T_1_ interconversion as a fast equilibrium can lead to noticeable error. Nevertheless, the fluorescence rate of ^2^T_1_ still exceeds the phosphorescence rate of ^4^T_1_ by three orders of magnitude, which more than compensates for the low steady state concentration of ^2^T_1_. Similar conclusions could be derived from the rates reported in Ake and Gouterman (1969) (3.6 × 10^3^s^−1^ and 8.3 × 10^−1^s^−1^, respectively), calculated from semiempirical exchange and SOC integrals and experimental absorption oscillator strengths, which agree surprisingly well with the rates that we obtained here. Kinetic simulation suggests that 99.2% of the total luminescence at this temperature is contributed by fluorescence, and only 0.8% is due to phosphorescence. This can be compared with the experimental finding by Bohandy and Kim (1980) that the phosphorescence of CuP at 86 K is observable as a minor 0–0 peak besides the 0–0 fluorescence peak, with a fluorescence to phosphorescence ratio of about 5:1 to 10:1 [as estimated from Figure 5 of Bohandy and Kim (1980)]; however note that this study was performed in a triphenylene solid matrix.

The total luminescence quantum yield is predicted by our kinetic simulations to be 5.3 × 10^−6^, four orders of magnitude smaller than the experimental quantum yield (0.09) in solution. We believe one possible reason is that the ^2^T_1_–^4^T_1_ gap of CuP is larger in solution than in the gas phase. This can already be seen from the experimental ^2^T_1_–^4^T_1_ 0–0 gaps of CuP in solid matrices with different polarities: the 0–0 gap was measured in polymethylmethacrylate as 500 cm^−1^ (Smith and Gouterman, 1968), but 310–320 cm^−1^ in *n*-octane (Noort et al., 1976) and 267 cm^−1^ in triphenylene (Bohandy and Kim, 1980). Therefore, the 0–0 gap in the gas phase is probably smaller than 267 cm^−1^, and indeed, our X-TDA calculations predict an adiabatic ^2^T_1_–^4^T_1_ gap of 92 cm^−1^ in the gas phase. The larger ^2^T_1_–^4^T_1_ gap in solution compared to the gas phase is expected to introduce a Boltzmann factor of $\exp\left(-\left(E_{sol}-E_{gas}\right)/RT\right)$ to *k*_RISC_, while changing the other rates negligibly. Setting *E*_sol_ = 267 cm^−1^ and *E*_gas_ = 92 cm^−1^, we obtain a solution phase *k*_RISC_ of 9.02 × 10^5^s^−1^, from which kinetic simulations give a fluorescence-phosphorescence ratio of 8.6:1, in quantitative agreement with experiment (Bohandy and Kim, 1980). Setting *E*_sol_ = 500 cm^−1^ [as appropriate for the polar solvent used in Eastwood and Gouterman (1969)] gives *k*_RISC_ = 1.60 × 10^4^s^−1^, and a total luminescence quantum yield of 4.0 × 10^−5^, with 13% contribution from fluorescence and 87% from phosphorescence. The remaining discrepancy (∼×2200) of the experimental and calculated quantum yields can be attributed to the restriction of the molecular vibrations of CuP by the low temperature (and thus viscous) solvent, which is expected to suppress the IC process significantly.

Interestingly, U-TDA completely fails to reproduce the qualitative picture of Figure 4 and predicts a ^2^T_1_–^4^T_1_ adiabatic gap of the wrong sign (−276 cm^−1^), violating Hund’s rule. At first sight, this may seem surprising: since the U-TDA “tripdoublet state” is a mixture of the true tripdoublet state and the quartet state, the U-TDA ^2^T_1_ energy should lie in between the energies of the true ^2^T_1_ state and the ^4^T_1_ state, which means that the U-TDA ^2^T_1_–^4^T_1_ gap should be smaller than the X-TDA gap but still have the correct sign. However, the U-TDA ^2^T_1_ state is contaminated by the *M*_*S*_ = 1/2 component of the ^4^T_1_ state (Eq. 5), while a spin flip-up U-TDA calculation of the ^4^T_1_ state gives its *M*_*S*_ = 3/2 component. The two spin components obviously have the same energy in the exact non-relativistic theory and in all rigorous spin-adapted methods, but not in U-TDA, even when the ground state is not spin-contaminated (Li and Liu, 2012; Li and Liu, 2016b). This shows that the restoration of the degeneracy of spin multiplets by the random phase approximation (RPA) correction in X-TDA (Li and Liu, 2011) indeed leads to qualitative improvement of the excitation energies, instead of being merely a solution to a conceptual problem. It also shows that estimating the tripdoublet energy by extrapolating from the energies of the spin-contaminated tripdoublet and the quartet by, e.g., the Yamaguchi method (Soda et al., 2000) does not necessarily give a qualitatively correct estimate of the spin-pure tripdoublet energy. The inverted doublet-quartet gap introduces qualitative defects to the computed photophysics of CuP. Already when the doublet-quartet gap is zero, the Boltzmann factor is expected to raise the *k*_RISC_ to 9.24 × 10^7^s^−1^, reducing the ratio of phosphorescence in the total luminescence to 0.11%. Further raising the quartet to reproduce the U-TDA doublet-quartet gap will reduce the *k*_ISC_ to 1.42 × 10^6^s^−1^, which reduces the ratio of phosphorescence to 0.0007%. These values are obviously in much worse agreement with the experiments (Bohandy and Kim, 1980).

Finally, we briefly comment on the luminescence lifetimes. The luminescence of CuP is known to decay non-exponentially (Smith and Gouterman, 1968), so its luminescence lifetime can only be approximately determined. The luminescence lifetime of CuP has been determined as 400 *μ*s (Smith and Gouterman, 1968) at 80 K in polymethylmethacrylate, and a biexponential decay with lifetimes 155 and 750 *μ*s was reported (Eastwood and Gouterman, 1969) at 78 K in methylphthalylethylglycolate. The same references also reported that the luminescence lifetimes of CuOEP and CuTPP are also within the 50–800 *μ*s range. However, in a room temperature toluene solution the luminescence lifetimes of CuOEP and CuTPP were reported to be 115 and 30 ns, respectively (Liu et al., 1995), and a few nanoseconds in the gas phase (Ha-Thi et al., 2013). If we define the luminescence lifetime as the time needed for 1–1/*e* ≈ 63.2% of the luminescence to be emitted, then kinetic simulations from our X-TDDFT rate constants give a gas-phase luminescence lifetime of 5.7 ns at 83 K, which is much shorter than the low-temperature condensed phase results, but in reasonable agreement with the room-temperature solution phase and especially the gas-phase experimental results. However, using the ISC and RISC rate constants consistent with the U-TDA doublet-quartet gap, one obtains a lifetime of 2.4 ns, differing noticeably from the X-TDDFT result. Thus, our results suggest that X-TDDFT/X-TDA gives non-negligible corrections upon the luminescence lifetime of U-TDDFT/U-TDA, and also confirm that the discrepancy of the experimental and calculated quantum yields is probably due to suppression of the IC of ^2^T_1_ by the low temperature solvent.

### 3.4 Discussions

**FIGURE 5:**
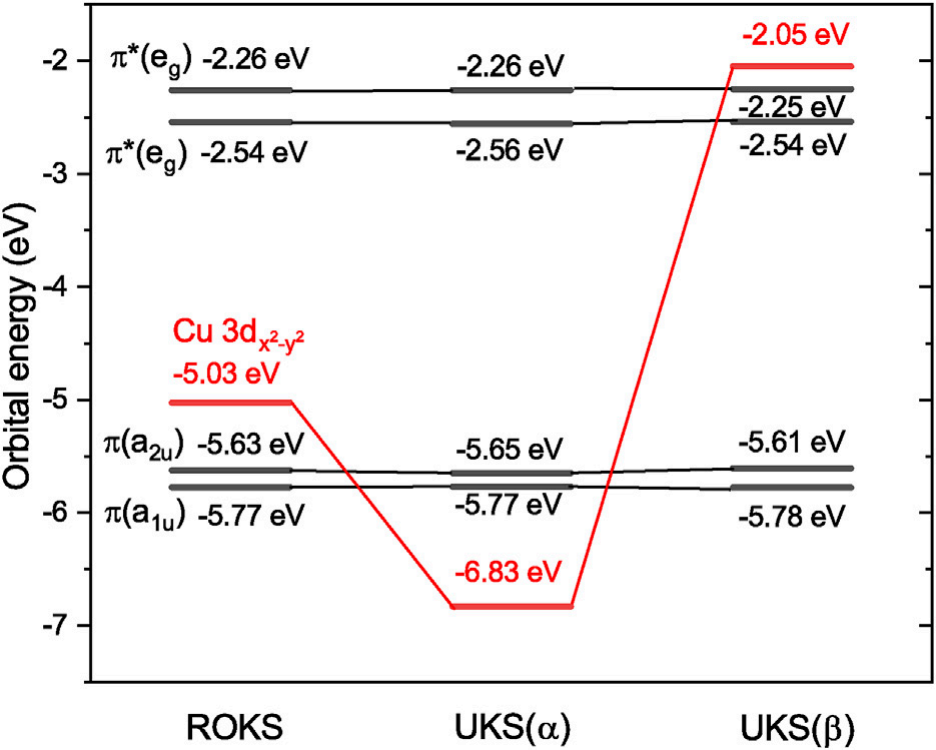
ROKS and UKS orbital energies of CuP at the X-TDDFT and U-TDDFT ^2^T_1_ equilibrium geometries, respectively, computed at the sf-X2C-PBE0/x2c-SVPall level of theory.

As mentioned in the Introduction, the simple orbital energy difference model based on a restricted open-shell determinant (Figure 1) predicts that the excitation energy of the lowest tripdoublet of any doublet molecule is at least the sum of the excitation energies of the first two excited states (as long as the ROKS ground state satisfies the *aufbau* rule). It therefore comes as a surprise that the lowest tripdoublet state of CuP (^2^T_1_) is only barely higher than the lowest excited state (^2^dd_1_) by 0.34 eV (Table 1), even though the ROKS ground state of CuP is indeed an *aufbau* state (Figure 5). This suggests a failure of the ROKS orbital energy difference model.

To understand why the ROKS orbital energies fail qualitatively for describing the excited state ordering of CuP, despite that the X-TDDFT method (which uses the ROKS determinant as the reference state) still gives reasonable excitation energies as compared to SDSPT2, we note that the *α* and *β* Fock matrices of an ROKS calculation are in general not diagonal under the canonical molecular orbital (CMO) basis. Only the unified coupling operator **R**, assembled from blocks of the CMO Fock matrices,

$$R=\left(\begin{array}{ccc} \frac{1}{2}\left(F_{\text{CC}\alpha }+F_{\text{CC}\beta }\right) & F_{\text{CO}\beta } & \frac{1}{2}\left(F_{\text{CV}\alpha }+F_{\text{CV}\beta }\right)\\ F_{\text{OC}\beta } & \frac{1}{2}\left(F_{\text{OO}\alpha }+F_{\text{OO}\beta }\right) & F_{\text{OV}\alpha }\\ \frac{1}{2}\left(F_{\text{VC}\alpha }+F_{\text{VC}\beta }\right) & F_{\text{VO}\alpha } & \frac{1}{2}\left(F_{\text{VV}\alpha }+F_{\text{VV}\beta }\right) \end{array}\right),$$ (6)

Is diagonal (Hirao and Nakatsuji, 1973). Note that herein we have used the Guest-Saunders parameterization (Guest and Saunders, 1974) of the diagonal blocks of **R**, which is the default choice of the BDF program, although our qualitative conclusions are unaffected by choosing other parameterizations. However, the leading term of the X-TDDFT calculation is not simply given by the eigenvalue differences of **R**,

$$\Delta_{ia\sigma ,jb\tau }^{\prime }=\delta_{\sigma \tau }\delta_{ij}\delta_{ab}\left(R_{aa}-R_{ii}\right),$$ (7)

but rather from the *α* and *β* Fock matrices themselves via

$$\Delta_{ia\sigma ,jb\tau }=\delta_{\sigma \tau }\left(\delta_{ij}F_{ab\sigma }-\delta_{ab}F_{ji\sigma }\right).$$ (8)

Here *i*, *a* represent occupied CMOs, *j*, *b* virtual CMOs, and *σ*, *τ* spin indices. For the diagonal matrix element of an arbitrary single excitation, Eqs 7, 8 differ by the following term:

$$\Delta_{ia\sigma ,ia\sigma }-\Delta_{ia\sigma ,ia\sigma }^{\prime }=\frac{1}{2}\left(\left(F_{aa\sigma }-F_{aa\sigma^{\prime }}\right)-\left(F_{ii\sigma }-F_{ii\sigma^{\prime }}\right)\right),$$ (9)

where *σ*′ is the opposite spin of *σ*. For a general hybrid functional, the Fock matrix element differences in Eq. 9 are given by (where *p* is an arbitrary CMO, *c*_x_ is the proportion of HF exchange, and *v* ^xc^ is the XC potential)

$$F_{pp\beta }-F_{pp\alpha }=c_{x}\left(pt|pt\right)+\left(v_{pp\beta }^{xc}-v_{pp\alpha }^{xc}\right),$$ (10)

Assuming, for the sake of simplicity, that there is only one open-shell orbital *t* in the reference state. Assuming that the XC potential behaves similarly as the exact exchange potential, the difference Eq. 10 is positive, and should usually be the largest when *p* = *t*, while being small when *p* is spatially far from *t*. The corollary is that the orbital energy difference approximation Eq. 7 should agree well with the X-TDDFT leading term Eq. 8 for CV excitations (where the difference is proportional to the small exchange integral (*pt*|*pt*)), but underestimate the excitation energies of CO and OV excitations by a correction proportional to the large (*tt*|*tt*) integral.

The underestimation of CO and OV excitation energies by ROKS orbital energy differences opens up the possibility of engineering a system to break the *ω*_*ia*_ = *ω*_*it*_ + *ω*_*ta*_ constraint inherent in the ROKS orbital energy difference model, and make the tripdoublet state the lowest excited state or only slightly higher than the lowest excited state. Possible approaches include:

1. Increase the difference Eq. 9 for the CO and OV states, while keeping it small for the lowest CV state, so that all CO and OV states are pushed above the lowest CV state. This is most easily done by making the open-shell orbital *t* very compact, which naturally leads to a larger *F*_*ttβ*_ − *F*_*ttα*_ (due to a larger (*tt*|*tt*)) but a smaller *F*_*ppβ*_ − *F*_*ppα*_, *p* ∈ {*i*, *a*} (due to a small absolute overlap between the *p* and *t* orbitals).
2. Reduce the orbital energy gap between the highest doubly occupied orbital and the lowest unoccupied orbital, which also helps to reduce the excitation energy of the lowest CV state. However, a too small orbital energy gap will favor the IC of the tripdoublet to the ground state, which may quench the fluorescence of the tripdoublet. As already mentioned in Section 3.3, the IC rate of CuP is already large enough to make CuP only barely fluorescent (quantum yield $<10^{-5}$ ) in the gas phase, and a viscous solvent seems to be required to suppress the IC contribution and make the fluorescence stronger.

**FIGURE 6:**
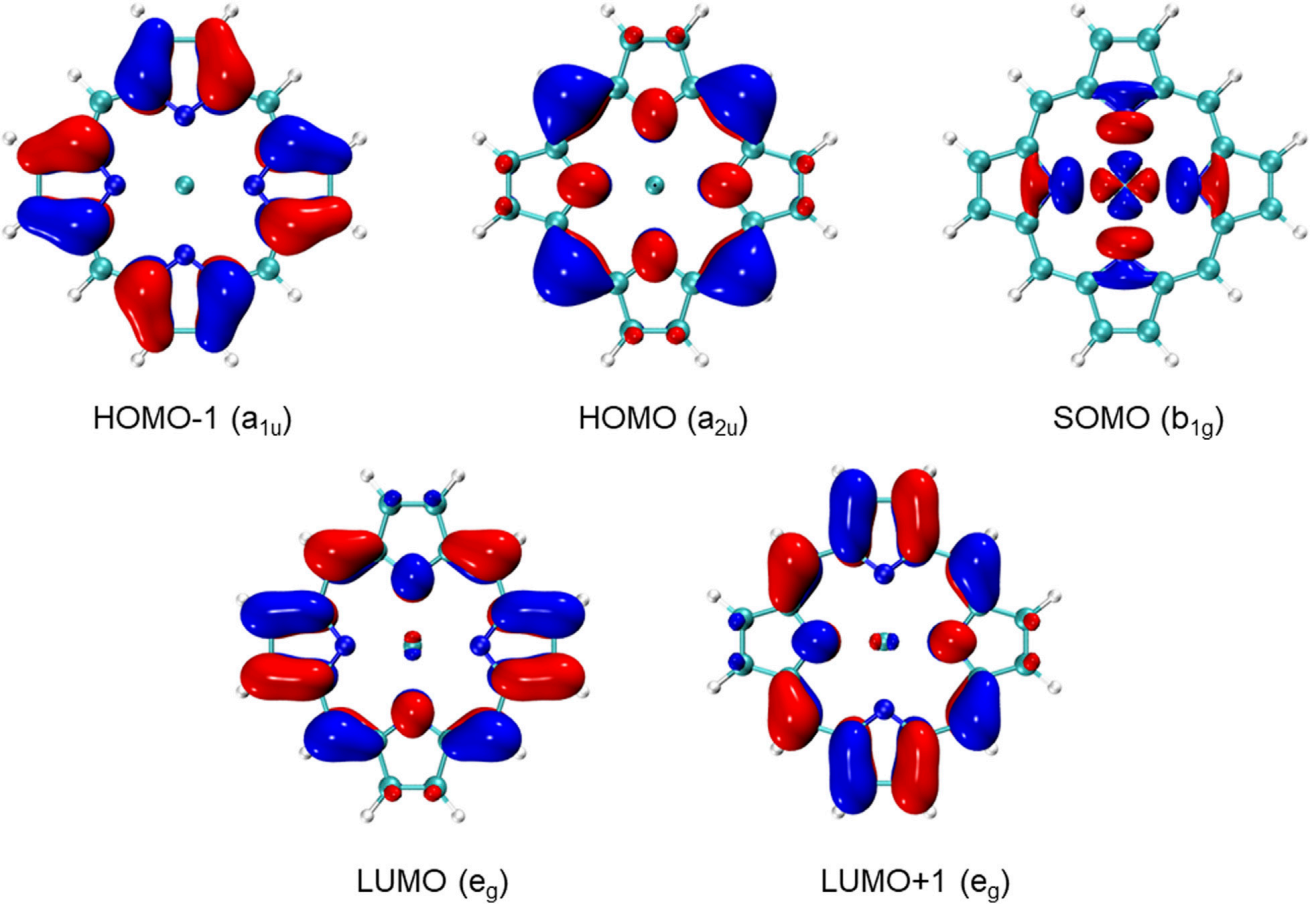
ROKS frontier molecular orbitals of CuP, computed at the sf-X2C-PBE0/x2c-SVPall level of theory.

Now, it becomes evident that CuP fits the above design principles very well. The unpaired electron in the ground state of CuP is on the Cu 3 $d_{x^{2}-y^{2}}$ orbital (Figure 6), which is spatially localized. Moreover, the Cu 3 $d_{x^{2}-y^{2}}$ orbital occupies a different part of the molecule than the ligand *π* and π* orbitals, which results in a small absolute overlap between the orbitals and helps to reduce the effect of Eq. 9 on the CV excitation energies. To quantitatively assess the effect of Eq. 9 on the CO and OV excitation energies, we note that the X-TDDFT leading term Eq. 8 is nothing but the UKS orbital energy difference, if the shape differences of the UKS and ROKS orbitals are neglected. Therefore, we have plotted the UKS orbital energies of CuP in Figure 5 as well. Intriguingly, the *α* Cu 3 $d_{x^{2}-y^{2}}$ orbital now lies below the porphyrin *π*(a_1*u*_) and *π*(a_2*u*_) orbitals, while the *β* Cu 3 $d_{x^{2}-y^{2}}$ orbital lies above the porphyrin π*(e_*g*_) orbitals. Therefore, the differences of UKS orbital energies predict that the lowest excited states of CuP are the CV states obtained from exciting an electron from *π*(a_1*u*_) and *π*(a_2*u*_) to π*(e_*g*_). This is not only consistent with our U-TD-PBE0 excitation energies, but also the X-TD-PBE0 results (save for the ^2^S_2_ state, which is higher than ^2^CT_1_ computed at the respective levels of theory), despite that the latter method is spin-adapted. Note also that although the (*tt*|*tt*) integral leads to a huge splitting between the *α* and *β* Cu 3 $d_{x^{2}-y^{2}}$ orbitals, the splitting is only barely enough for the UKS orbital energy differences to predict a tripdoublet first excited state: if the *β* Cu 3 $d_{x^{2}-y^{2}}$ orbital were just 0.2 eV lower, one would predict that the CO-type CT excitation *π*(a_2*u*_) → Cu 3 $d_{x^{2}-y^{2}}$ is lower than the lowest tripdoublet *π*(a_2*u*_) → π*(e_*g*_). Alternatively, one may say that the HOMO-LUMO gap of the porphyrin ligand is barely narrow enough to fit within the energy window between the *α* and *β* Cu 3 $d_{x^{2}-y^{2}}$ orbitals, which clearly illustrates the importance of using a narrow-gap ligand for designing systems with a low-lying tripdoublet excited state.

Besides, we note that our SDSPT2 vertical absorption energy results suggest that three d-d excited states, ^2^dd_1_, ^2^dd_2_ and ^2^dd_3_, lie barely below the ^2^T_1_ state, although the actual energy ordering of ^2^dd_3_ and ^2^T_1_ cannot be said with full certainty. This means that CuP undergoes anti-Kasha fluorescence, which (as mentioned in the Introduction) is difficult to achieve especially for open-shell molecules. The ^2^dd states are very close in energy to the ^2^T_1_ state, strongly favoring an ultrafast IC process, while the ^2^dd states themselves relax radiationlessly to the ground state on the hundred picosecond timescale, mainly through the cascade ^2^dd_2_ →^2^dd_1_ →^2^S_0_ (Figure 4). In reality, however, our calculations suggest a ^2^T_1_ →^2^dd_1_ IC rate of only 2.00 × 10^8^s^−1^ (with other ^2^T_1_ →^2^dd IC processes even slower), which although competitive with the ^2^T_1_ →^2^S_0_ IC rate, is very small for an IC process with an adiabatic gap of only 0.42 eV (the estimated SDSPT2 adiabatic excitation energies of the ^2^dd_1_, ^2^dd_2_, ^2^dd_3_ and ^2^T_1_ being 1.66, 1.90, 2.06 and 2.08 eV, respectively). The reason is that the ^2^T_1_ and ^2^dd states differ by a double excitation, while the nuclear derivative operator in the NACMEs is a one-electron operator, making the IC forbidden to first order. Moreover, to transform the ^2^T_1_ state to any of the ^2^dd states, one has to perform an LMCT excitation as well as an MLCT excitation (from α π*(e_*g*_) to *α* Cu 3 $d_{x^{2}-y^{2}}$ , and from *β* Cu 3d_*xy*_/3d_*xz*_/3d_*yz*_/3 $d_{z^{2}}$ to *β π*(a_2*u*_)), both of which involve occupied/virtual orbital pairs with poor overlap. Therefore, the drastically different natures of the ligand-centered ^2^T_1_ state and the metal-centered ^2^dd states effectively prevented the quenching of the luminescence by the ^2^dd states, despite that the ^2^T_1_ and ^2^dd states are extremely close in energy.

To conclude this section, we briefly note that even making the first doublet excited state a tripdoublet state still does not guarantee the realization of tripdoublet fluorescence. Two remaining potential obstacles are (1) the IC of the tripdoublet state to the ground state and (2) the ISC of the tripdoublet to the lowest quartet state (which is almost always lower than the lowest tripdoublet state owing to Hund’s rule). Both can be inhibited by making the molecule rigid, which is indeed satisfied by the porphyrin ligand in CuP. Alternatively, if the ISC from the first quartet state to the ground state is slow (as is the case of CuP, thanks to the spatial symmetry selection rules), and the gap between the first doublet and the first quartet is comparable to the thermal energy *kT* at the current temperature, then the quartet state can undergo RISC to regenerate the tripdoublet state, which can then fluoresce. This is well-known as the thermally activated delayed fluorescence (TADF) mechanism (Parker and Hatchard, 1961; Endo et al., 2011; Yang et al., 2017), although existing TADF molecules typically fluoresce from singlets and use a triplet “reservoir state” to achieve delayed fluorescence. In order for the TADF mechanism to outcompete the phosphorescence from the first quartet state, both the phosphorescence rate and the doublet-quartet gap have to be small. While the low phosphorescence rate of CuP can be explained by the fact that copper is a relatively light element, the small ^2^T_1_–^4^T_1_ gap of CuP can be attributed to the distributions of the frontier orbitals of CuP. Recall that the X-TDDFT gap between a tripdoublet excitation Eq. 3 and the associated quartet excitation Eq. 4 is exactly given by the X-RPA gap (Li and Liu, 2011), which is equal to $\frac{3}{2}\left(\left(it|it\right)+\left(ta|ta\right)\right)$ . However, both of the two integrals are small for the ^2^T_1_ and ^4^T_1_ states of CuP, since the orbitals *i* and *a* reside on the ligand while *t* is localized near the metal atom (Figure 6). Such a clean spatial separation of the metal and ligand CMOs (despite the close proximity of the metal and the ligand) can further be attributed to the fact that the Cu 3 $d_{x^{2}-y^{2}}$ orbital has a different irrep than those of the ligand *π* and π* orbitals, preventing the delocalization of the open-shell orbital to the *π* system of the porphyrin ligand; while the Cu 3 $d_{x^{2}-y^{2}}$ orbital can still delocalize through the *σ* bonds of the ligand, the delocalization is of limited extent due to the rather local electronic structures of typical *σ* bonds (Figure 6). Incidentally, the only other class of tripdoublet-fluorescing metalloporphyrins that we are aware of, i.e., vanadium(IV) oxo porphyrin complexes (Ake and Gouterman, 1969; Gouterman et al., 1970), are characterized by a single unpaired electron in the 3d_*xy*_ orbital, whose mixing with the ligand *π* and π* orbitals is also hindered by symmetry mismatches. Whether this can be extended to a general strategy of designing molecules that fluoresce from tripdoublet states (or more generally, molecules that possess small doublet-quartet gaps) will be explored in the future. Finally, we briefly note that the design of doublet molecules with TADF and/or phosphorescence is also an interesting subject and deserves attention in its own right.

## 4 Conclusion

Fluorescence of open-shell molecules from tripdoublet states is a rare and underexplored phenomenon, for which traditional excited state methods such as U-TDDFT are unreliable due to severe spin contamination. In this work, we employed the high-precision method SDSPT2 to obtain accurate excitation energies of the CuP molecule, which suggests that the bright states obtained by light absorption relax to a low-lying doublet state, ^2^T_1_, via a cascade of ultrafast IC processes, in agreement with experiments. ^2^T_1_ is a tripdoublet state composed of a triplet ligand state antiferromagnetically coupled with the unpaired electron of Cu(II), and contrary to predictions from ROKS orbital energy differences, the only excited states lower than ^2^T_1_ are the d-d excited states ^2^dd_1_, ^2^dd_2_ and ^2^dd_3_, and the nonradiative relaxations from ^2^T_1_ to the ^2^dd states are slower than expected from their energy gaps, due to the large differences in excited state compositions. Using the SDSPT2 results as a benchmark, we found that the X-TDDFT method provides a more accurate description of the ^2^T_1_ state (which exhibits considerable spin contamination) compared to U-TDDFT, while for the CO excitations, U-TDDFT and X-TDDFT show similar performance.

In addition to vertical absorption calculations and structural analyses, we conducted a detailed analysis of the relaxation rate constants of the excited states of CuP. Our results suggest that, in the gas phase and at low temperature (83 K), CuP emits fluorescence from the lowest tripdoublet state ^2^T_1_ with a very small quantum yield ( $∼10^{-5}$ ), and the contribution of phosphorescence is negligible. These results complement the experimental results in solution phase and solid matrix, which gave a lower but still greater than unity fluorescence-to-phosphorescence ratio and a much higher luminescence quantum yield. The discrepancies are nicely explained by a solvent-dependent ^2^T_1_–^4^T_1_ gap and the viscosity of the solvent. Furthermore, we confirm the presence of an equilibrium between the first doublet state ^2^T_1_ and the first quartet state ^4^T_1_, the latter of which functions as a reservoir of the ^2^T_1_ state, although the steady state concentration ratio of these two states deviates noticeably from their equilibrium constant. CuP therefore represents an interesting example of a TADF molecule that emits fluorescence through a doublet-doublet transition, instead of the much more common singlet-singlet pathway. Notably, U-TDA predicts a doublet-quartet gap of the wrong sign, due to the spin contamination of the doublet state as well as the breaking of the spin multiplet degeneracy of the quartet state. Although the error is small ( < 0.05 eV), it translates to a large error in the luminescence lifetime and (even more) the contribution of phosphorescence to the total luminescence. This again highlights the importance of using spin-adapted approaches in the study of open-shell systems, even when the excitation energy errors of unrestricted methods are small.

Based on the computational results, we proposed a few possible approaches that can be used to design new doublet molecules that fluoresce from tripdoublets: 1) keep the open-shell orbital of the molecule spatially compact, to open up a gap between the *α* and *β* UKS orbital energies of the open-shell orbital; 2) make the gap between the highest doubly occupied orbital and the lowest vacant orbital small enough so that both orbitals fit into the gap between the *α* and *β* open-shell orbitals, but not overly small as to encourage IC of the lowest tripdoublet state to the ground state; 3) make the molecule rigid to minimize unwanted non-radiative relaxation processes; 4) avoid introducing heavy elements in order to suppress unwanted ISC and phosphorescence processes; 5) localize the open-shell orbital and the frontier *π*/π* orbitals onto different molecular fragments, and (if possible) make them belong to different irreps, to minimize the doublet-quartet gap; and 6) when the presence of doublet excited states below the tripdoublet is unavoidable, make those states differ significantly in composition from the tripdoublet state, to avoid the quenching of tripdoublet fluorescence by excited state-excited state IC. We hope that the present work will facilitate the discovery of novel molecules that fluoresce from tripdoublet states. Moreover, we expect that the success of the X-TDDFT and SDSPT2 methods will encourage the use of these two methods in the excited state studies of other systems.

## Data Availability

The original contributions presented in the study are included in the article, further inquiries can be directed to the corresponding authors.
